# Clinical implication of minimal presence of solid or micropapillary subtype in early‐stage lung adenocarcinoma

**DOI:** 10.1111/1759-7714.13754

**Published:** 2020-11-24

**Authors:** Sun Ha Choi, Ji Yun Jeong, Shin Yup Lee, Kyung Min Shin, Shin Young Jeong, Tae‐In Park, Young Woo Do, Eung Bae Lee, Yangki Seok, Won Kee Lee, Ji Eun Park, Sunji Park, Yong Hoon Lee, Hyewon Seo, Seung Soo Yoo, Jaehee Lee, Seung‐Ick Cha, Chang Ho Kim, Jae Yong Park

**Affiliations:** ^1^ Department of Internal Medicine, School of Medicine Kyungpook National University Daegu Korea; ^2^ Lung Cancer Center Kyungpook National University Chilgok Hospital Daegu Korea; ^3^ Department of Pathology, School of Medicine Kyungpook National University Daegu Korea; ^4^ Vessel‐Organ Interaction Research Center Kyungpook National University Daegu Korea; ^5^ Department of Radiology, School of Medicine Kyungpook National University Daegu Korea; ^6^ Department of Nuclear Medicine, School of Medicine Kyungpook National University Daegu Korea; ^7^ Department of Thoracic Surgery, School of Medicine Kyungpook National University Daegu Korea; ^8^ Department of Thoracic Surgery Soonchunhyang University Gumi Hospital Gumi Korea; ^9^ Medical Research Collaboration Center in Kyungpook National University Hospital and School of Medicine Kyungpook National University Daegu Korea

**Keywords:** Lung adenocarcinoma, micropapillary, prognosis, solid, stage IA

## Abstract

**Background:**

We investigated the clinical features and surgical outcomes of lung adenocarcinoma with minimal solid or micropapillary (S/MP) components, with a focus on stage IA.

**Methods:**

We enrolled 506 patients with lung adenocarcinoma who underwent curative resection in this study. Clinical features and surgical outcomes were compared between the groups with and without the S/MP subtype (S/MP+ and S/MP−, respectively), and between the group with an S/MP proportion of ≤5% (S/MP5) and the S/MP−.

**Results:**

The S/MP subtype was present in 247 patients (48.8%); 129 (25.5%) were grouped as the S/MP5 group. The S/MP+ and S/MP5 groups had larger tumors, higher frequency of lymph node metastasis, and more advanced stages of disease than the S/MP− group (*P* < 0.001, all comparisons). Pleural, lymphatic, and vascular invasions occurred more frequently in the S/MP+ and S/MP5 groups (*P* < 0.001, all comparisons for S/MP+ vs. S/MP−; *P* ≤ 0.01, all comparisons for S/MP5 vs. S/MP−). The S/MP+ and S/MP5 groups showed a shorter time to recurrence and cancer‐related death than the S/MP− group(*P* < 0.001, both comparisons). For stage I, the presence or absence of the S/MP subtype defined prognostic subgroups better than the stage IA/IB classification. Notably, in the multivariate analysis, the minimal S/MP component was a significant predictor of recurrence, even in stage IA.

**Conclusions:**

The presence of the minimal S/MP component was a significant predictor of poor prognosis after surgery, even in stage IA patients. Clinical trials to evaluate the advantages of adjuvant chemotherapy for this subset of patients and further investigations to understand underlying biological mechanisms of poor prognosis are needed.

**Key points:**

Significant findings of the study: We demonstrated that only minimal presence of solid or micropapillary component was profoundly associated with aggressive clinicopathological features and poor prognosis after complete resection even in stage IA lung adenocarcinoma. What this study adds: Our results suggest that minimal presence of these subtypes is a strong prognostic factor which should be taken into account in the risk assessment for adjuvant chemotherapy in lung adenocarcinoma.

## Graphical abstract

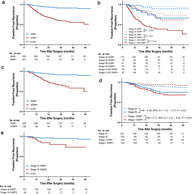

## Introduction

Surgical resection offers the best hope of a cure in patients with early‐stage non‐small cell lung cancer (NSCLC). However, the recurrence rate after complete resection remains high. Postoperative adjuvant chemotherapy has proven beneficial to NSCLC patients in clinical trials.^1^, ^2^ Based on the eighth edition of the TNM classification,^3^ adjuvant chemotherapy is generally recommended for patients with stage IIB or higher disease, and may be considered in patients with T2a/bN0 or stages IB/IIA in the presence of high‐risk factors such as poorly differentiated tumor, vascular invasion, and visceral pleural invasion among others.^4^ For stage IA NSCLC patients, there is currently no evidence to support the use of adjuvant chemotherapy. However, a substantial proportion of stage IA patients experience recurrence and subsequent death after complete resection, which presents an important unmet need.

A new histopathological classification was proposed by the World Health Organization in 2015^5^ based on the 2011 International Association for the Study of Lung Cancer/American Thoracic Society/European Respiratory Society (IASLC/ATS/ERS) classification.^6^ The classification recommends semiquantitative recording, in 5% increments, of all subtypes of resected invasive lung adenocarcinoma, including lepidic, acinar, papillary, solid (S), and micropapillary (MP) patterns, as well as the determination of the predominant subtype and additional minor components.^6^ Several studies have shown that the classification provides prognostic information; S or MP (S/MP) subtype‐predominant tumors have been associated with earlier recurrence and worse post‐resection survival and are therefore categorized as high grade.^7^, ^8^ In addition, when the S/MP subtype was found to be present as a minor subtype, such as a nonpredominant S/MP subtype, it also correlated with a worse prognosis.^9^, ^10^, ^11^, ^12^ Some studies evaluated the unfavorable prognostic effect of the S/MP subtype by comparing the outcomes between the presence and absence of these subtypes.^10^, ^11^, ^12^ Nonetheless, because the presence of S/MP subtype in these studies stood for a wide range of S/MP proportions from minimal to predominant percentages, it remains unclear whether the minimal presence of the S/MP subtype has a clinically significant effect on the surgical outcomes.

This study aimed to evaluate the clinicopathological characteristics and surgical outcomes of lung adenocarcinoma with minimal S/MP subtype component. In particular, we investigated whether the minimal presence of the S/MP subtype could define a subset of patients at an increased risk of recurrence after surgery who may benefit from adjuvant chemotherapy in stage IA lung adenocarcinoma.

## Methods

### Patients

We undertook a retrospective review of patient medical records and pathological reports, and enrolled 506 patients with pathological stage I, II, or IIIA invasive adenocarcinoma of the lung. All patients underwent curative surgical resection at Kyungpook National University Chilgok Hospital (KNUCH) in Daegu, Korea between January 2014 and June 2019. The majority of patients with clinical stage IIIA disease received concurrent chemoradiation at KNUCH; only patients who were postoperatively found to have stage IIIA disease with microscopic N2 metastasis were included in this study. We excluded patients who received neoadjuvant treatment followed by surgery because of the effect of chemotherapy or radiotherapy on the tumor pathology. All patients were restaged in accordance with the eighth TNM classification. Lobectomy combined with systemic lymph node dissection through thoracotomy or video‐assisted thoracic surgery was the standard surgical procedure at KNUCH. The current study included 503 lobectomy cases and three bilobectomy cases, and excluded patients who underwent limited resection. Only patients who received R0 resection were included. Postoperatively, the patients were intensively followed‐up by history taking, physical examination, and chest/upper abdominal computed tomography (CT) every four months in the first two years and every six months thereafter until the end of the fifth year. Additional evaluations, including bone scan, PET/CT, and laboratory investigations, were conducted if necessary. The study protocol was approved by the institutional review board (approval No. KNUCH 2019‐04‐014) and the need for informed consent was waived in consideration of the retrospective nature of the evaluation of anonymized medical data.

### Histological evaluation

For histological evaluation, 10% formalin‐fixed and paraffin‐embedded tumor sections were cut and stained with hematoxylin and eosin. Comprehensive histological subtyping based on the 2011 IASLC/ATS/ERS classification was performed at the time of pathological diagnosis of surgical specimens (J.Y.J) and reviewed by two experienced pathologists (J.Y.J. and T.I.P) prior to analysis; disagreements, if any, were resolved through discussion, and a consensus was reached using a multihead microscope. All subtypes of resected invasive lung adenocarcinoma were categorized as lepidic, acinar, papillary, S, or MP and the presence of each subtype component was recorded in 5% increments, to constitute a total of 100% of subtype components per tumor. The pattern with the largest percentage was defined as the predominant pattern. A subtype component was considered to be present when it occupied 1% or more of the entire tumor. All pathology reports were completed after reviewing slides as thoroughly as possible. Small masses less than 2–3 cm were cut at every 3–5 mm to create paraffin blocks, and a whole‐tissue section made from each block could be placed on a single slide. Therefore, the pathologists could actually evaluate almost every part of the mass by examining 5–6 slides per mass. For larger masses, the pathologists usually examined 5–6 different parts of the masses, and the average percentage of each subtype component was recorded in the pathology reports. For the analysis, we grouped tumors in which the S or MP subtypes were present as S/MP+, whereas tumors without both S and MP were grouped as S/MP−. Tumors with 5% or less of S/MP subtype were grouped as S/MP5.

### Statistical analysis

Data were expressed as medians with ranges for continuous variables, and as numbers with percentages for categorical variables. We used the Mann‐Whitney U test for comparisons of continuous variables, and the chi‐square or Fisher's exact test for comparisons of categorical variables. We defined time to recurrence (the duration of freedom from recurrence [FFR]) as the interval from surgery to the first evidence of disease recurrence or the last evaluation; the duration of disease‐specific survival (DSS) was calculated from the date of surgery until the date of cancer‐related death or final follow‐up. Data were censored at the final follow‐up when the patient was alive without recurrence, or when the patient had died without recurrence. Kaplan‐Meier analyses and log‐rank tests were used to evaluate the differences in FFR and DSS. The Cox proportional hazard model was used for multivariate survival analyses. The hazard ratio (HR) and 95% confidence interval (CI) were estimated. All tests for significance were two‐sided, and all variables with a *P*‐value of less than 0.05 were considered statistically significant. All statistical analyses were undertaken using SPSS version 25.0 (IBM Corporation, Armonk, NY, USA) and GraphPad Prism 8 (GraphPad Software, San Diego, CA, USA).

## Results

The S/MP subtype was present in 48.8% (247/506) of patients. Overall, 9.9% (50/506) of patients had S‐predominant tumors, 0.2% (1/506) had MP‐predominant tumors, and 25.5% (129/506) had an S/MP proportion of 5% or less (Table 1). The S/MP subtype was significantly associated with age (*P* = 0.05), male sex (*P* < 0.001), and smoking (*P* < 0.001). The tumor size was significantly larger in the S/MP+ group (2.5 [1.0–10.0] cm) than in the S/MP− group (1.8 [0.6–9.0] cm, *P* < 0.001). Patients in the S/MP+ group had lymph node metastasis more frequently (28.3% vs. 4.6%, *P* < 0.001) and were diagnosed at more advanced stages (*P* < 0.001) than those in the S/MP− group. Pleural (44.5% vs. 21.6%), lymphatic (25.9% vs. 5.8%), and vascular (12.6% vs. 2.3%) invasions were more frequent in the S/MP+ group (*P* < 0.001 for all comparisons). Acinar and lepidic subtype‐predominant tumors were significantly associated with the S/MP− group (*P* < 0.001 for both comparisons). The frequency of *EGFR* mutation, *ALK* rearrangement, and positive PD‐L1 expression (≥1%) did not differ between the S/MP+ and the S/MP− groups (41.2% vs. 45.2%, *P* = 0.42; 6.1% vs. 2.9%, *P* = 0.12; 83.8% vs. 75.0%, *P* = 0.22, respectively). However, high PD‐L1 expression (≥50%) was more frequent in the S/MP+ group than in the S/MP− group (32.4% vs. 15.4%, *P* = 0.03). With regard to CT features, pure ground‐glass nodules and part‐solid nodules were found significantly more frequently in the S/MP− group than in the S/MP+ or S/MP5 groups (*P* < 0.001 for all comparisons), whereas solid nodules occurred significantly more frequently in the S/MP+ or S/MP5 groups than in the S/MP− group (*P* < 0.001 for both comparisons). In part‐solid nodules, a high consolidation‐to‐tumor ratio was significantly associated with the presence of the S/MP subtype (*P* = 0.01). These results suggest that solid component on CT is an important radiological factor for preoperative prediction of the S/MP subtype. PET/CT showed that S/MP+ had significantly higher ^18^F‐FDG uptake than S/MP− (SUVmax, 8.2 [1.3–45.1] vs. 2.8 [0.7–34.0], *P* < 0.001). Patients in the S/MP+ group received adjuvant chemotherapy more frequently than those in the S/MP− group (42.9% vs. 13.1%, *P* < 0.001).

**Table 1:** Clinicopathological characteristics according to solid or micropapillary subtype component

Characteristics	S/MP−*n* = 259 (51.2)	S/MP+*n* = 247 (48.8)	*P‐*value*	S/MP5*n* = 129 (25.5)	*P‐*value**
Age, years	64 (26–82)	65 (37–84)	0.05	67 (37–84)	0.06
Sex			<0.001		0.45
Female	157 (60.6)	109 (44.1)		73 (56.6)	
Male	102 (39.4)	138 (55.9)		56 (43.4)	
Smoking status^†^			<0.001		0.17
Never	170/257 (66.1)	112 /244 (45.9)		75/127 (59.1)	
Ever	87/257 (33.9)	132/244 (54.1)		52/127 (40.9)	
Pack‐years	35.0 (2.5–150.0)	35.0 (1.5–120.0)	0.90	33.0 (1.5–120.0)	0.87
Tumor size, cm	1.8 (0.6–9.0)	2.5 (1.0–10.0)	<0.001	2.4 (1.0–10.0)	<0.001
>3 cm	33 (12.7)	73 (29.6)	<0.001	32 (24.0)	0.005
N stage			<0.001		<0.001
N0	247 (95.4)	177 (71.7)		104 (80.6)	
N1	7 (2.7)	41 (16.6)		17 (13.2)	
N2	5 (1.9)	29 (11.7)		8 (6.2)	
Pathological stage			<0.001		<0.001
IA	173 (66.8)	78 (31.6)		48 (37.2)	
IB	61 (23.6)	72 (29.1)		46 (35.7)	
IIA	8 (3.1)	3 (1.2)		2 (1.6)	
IIB	12 (4.6)	59 (23.9)		23 (17.8)	
III	5 (1.9)	35 (14.2)		10 (7.8)	
Tumor invasion					
Pleural	56 (21.6)	110 (44.5)	<0.001	54 (41.9)	<0.001
Lymphatic	15 (5.8)	64 (25.9)	<0.001	24 (18.6)	<0.001
Vascular	6 (2.3)	31 (12.6)	<0.001	10 (7.8)	0.01
Lymphovascular	20 (7.7)	83 (33.6)	<0.001	32 (24.8)	<0.001
Predominant subtype					
Acinar	163 (62.9)	106 (42.9)	<0.001	68 (52.7)	0.05
Lepidic	17 (6.6)	1 (0.4)	<0.001	0	NE
Papillary	79 (30.5)	89 (36.0)	0.19	61 (47.3)	0.001
Solid	0	50 (20.2)	NE	0	NE
Micropapillary	0	1 (0.4)	NE	0	NE
Genetic mutation^†^					
*EGFR*	85/188 (45.2)	87/211 (41.2)	0.42	52/103 (50.5)	0.39
*ALK*	6/207 (2.9)	13/214 (6.1)	0.12	6/112 (5.4)	0.27
PD‐L1 expression^†^					
PD‐L1 ≥1%	39/52 (75.0)	62/74 (83.8)	0.22	26/33 (78.8)	0.69
PD‐L1 ≥50%	8/52 (15.4)	24/74 (32.4)	0.03	4/33 (12.1)	0.76
CT features					
Pure GGN	31 (12.0)	0 (0)	<0.001	0 (0)	<0.001
Part‐solid nodule	114 (44.0)	50 (20.2)	<0.001	32 (24.8)	<0.001
0 < CTR ≤ 0.55^‡^	54 (47.4)	13 (26.0)	0.01	11 (34.4)	0.19
0.55 < CTR < 1^‡^	60 (52.6)	37 (74.0)		21 (65.6)	
Solid nodule	114 (44.0)	197 (79.8)	<0.001	97 (75.2)	<0.001
SUVmax^†^	2.8 (0.7–34.0)	8.2 (1.3–45.1)	<0.001	6.9 (1.3–45.1)	<0.001
Adjuvant chemotherapy	33 (12.7)	106 (42.9)	<0.001	46 (35.7)	<0.001
Recurrence	18 (6.9)	77 (31.2)	<0.001	35 (27.1)	<0.001
Cancer‐related death	7 (2.8)	24 (10.0)	0.001	13 (10.1)	0.002

Data are presented as medians (range) or n (%).

*ALK*, anaplastic lymphoma kinase; CT, computed tomography; CTR, consolidation‐to‐tumor ratio; *EGFR*, epidermal growth factor receptor; GGN, ground‐glass nodule; PD‐L1, programmed death ligand 1; S/MP−, both solid and micropapillary subtype absent; S/MP+, solid or micropapillary subtype present; S/MP5, solid or micropapillary subtype proportion ≤ 5%; SUVmax, maximal standardized uptake value.

* *P*‐value for comparison between S/MP− and S/MP+.

** *P*‐value for comparison between S/MP− and S/MP5.

^†^ Values were calculated among patients for whom the data were available.

^‡^ Median CTR = 0.55.

To evaluate whether even the minimal presence of the S/MP subtype component was associated with aggressive features, we compared the S/MP5 group to the S/MP− group (Table 1). The S/MP5 group had larger tumors (2.4 vs. 1.8 cm, *P* < 0.001), higher frequency of lymph node metastasis (19.4% vs. 4.6%, *P* < 0.001), and more advanced stages of disease at diagnosis (*P* < 0.001) than the S/MP− group. Pleural (41.9% vs. 21.6%, *P* < 0.001), lymphatic (18.6% vs. 5.8%, *P* < 0.001), and vascular (7.8% vs. 2.3%, *P* = 0.01) invasions were more frequent in the S/MP5 than the S/MP− group. The S/MP5 group had a significantly higher SUVmax value than the S/MP− group (6.9 [1.3–45.1] vs. 2.8 [0.7–34.0], *P* < 0.001). More patients in the S/MP5 group received adjuvant chemotherapy (35.7% vs. 13.1%, *P* < 0.001).

**Figure 1:**
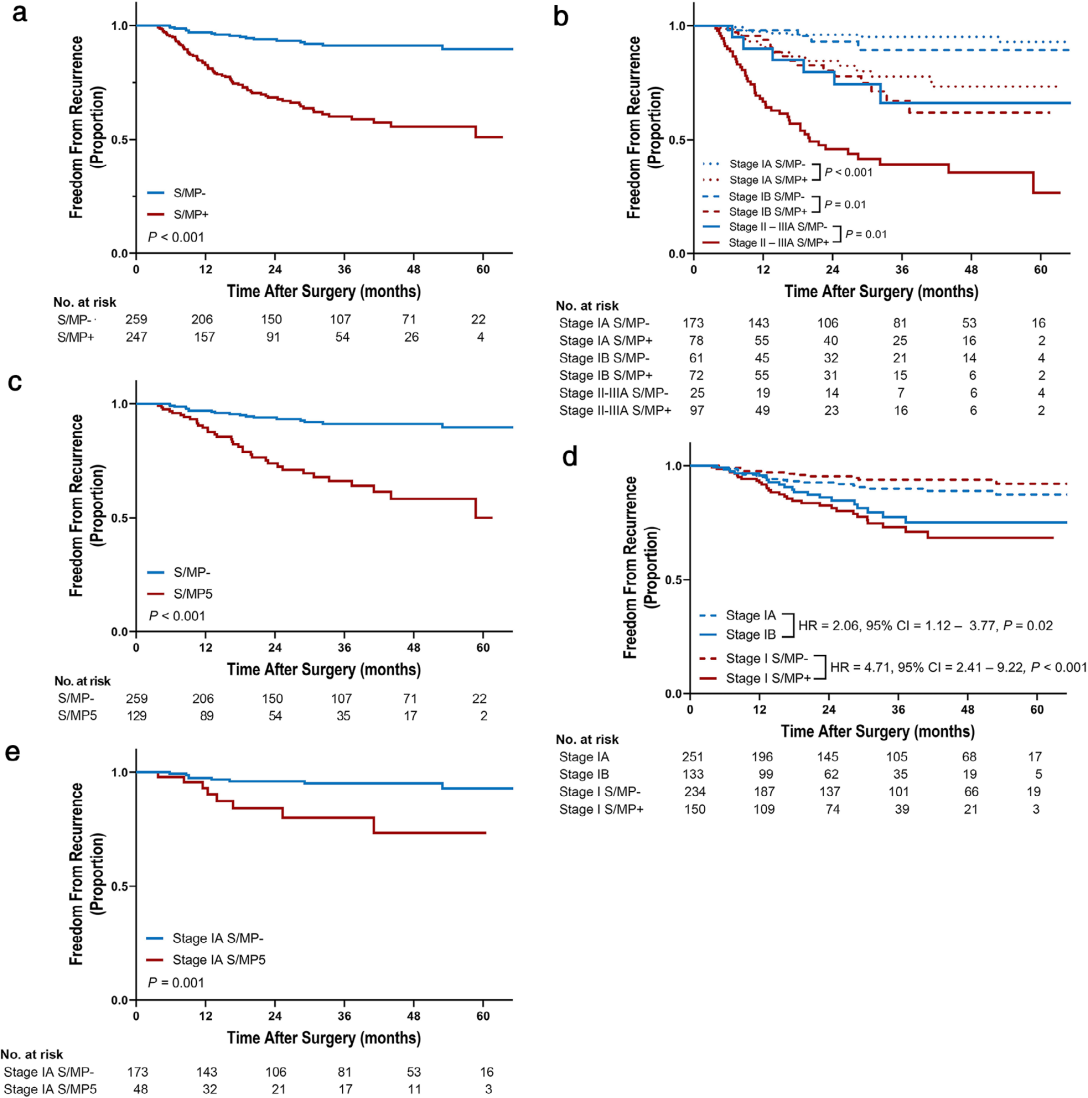
Kaplan‐Meier plots for freedom from recurrence according to S/MP subtype component. (**a**) S/MP+ vs. S/MP− 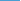, S/MP−; 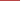, S/MP+, (**b**) S/MP+ vs. S/MP− in each stage 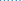, Stage IA S/MP−; 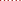, Stage IA S/MP+; 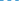, Stage IB S/MP−; 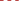, Stage IB S/MP+; 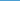, Stage II–IIIA S/MP−; 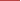, Stage II–IIIA S/MP+, (**c**) S/MP5 vs. S/MP− 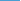, S/MP−; 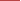, S/MP5, (**d**) Comparison of S/MP+ vs. S/MP− and IB vs. IA in stage I 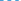, Stage IA; 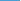, Stage IB; 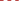, Stage I S/MP−; 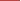, Stage I S/MP+, (**e**) S/MP5 vs. S/MP− in stage IA 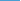, Stage IA S/MP−; 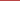, Stage IA S/MP5. MP, micropapillary; S, solid; S/MP+, solid or micropapillary subtype present; S/MP−, both solid and micropapillary subtype absent; S/MP5, solid or micropapillary subtype proportion of 5% or less. *P*‐values by log‐rank test and hazard ratio (HR) and 95% confidence interval (CI) estimated by univariate Cox model.

The median follow‐up duration was 32.8 (3.5–74.4) months. Overall, the S/MP+ group showed significantly worse FFR and worse DSS than the S/MP− group (Fig 1a and Fig S1a, *P* < 0.001 for both comparisons). In the subgroup analysis, the S/MP+ group had significantly worse FFR than the S/MP− group in each stage (*P* ≤ 0.01 for all comparisons, Fig 1b). Furthermore, the S/MP5 group had significantly worse FFR and worse DSS than the S/MP− group (Fig 1c and Fig S1b, *P* < 0.001 for both comparisons). Notably, the separation of low‐ and high‐risk groups for recurrence in stage I (*n* = 384) was more obvious based on the presence or absence of the S/MP subtype (stage I S/MP+ vs. stage I S/MP−, HR = 4.71, 95% CI: 2.41–9.22, *P* < 0.001) than based on tumor size (stage IB vs. stage IA, HR = 2.06, 95% CI: 1.12–3.77, *P* = 0.02; Fig 1d). In multivariate analyses, both the S/MP and S/MP5 were independent risk factors for recurrence (adjusted HR [aHR] = 3.10, 95% CI: 1.76–5.46, *P* < 0.001; aHR = 3.20, 95% CI: 1.73–5.90, *P* < 0.001, respectively, Table 2), along with tumor size, lymph node metastasis, and lymphovascular invasion.

**Table 2:** Univariate and multivariate analyses for freedom from recurrence after curative resection

Variables	Univariate analysis			Multivariate analysis		
	HR	95% CI	*P*‐value	HR	95% CI	*P*‐value
S/MP+ vs. S/MP−						
Age^†^	1.04	1.01–1.06	0.001	1.02	1.00–1.05	0.05
Male sex (vs. female)	1.33	0.89–1.99	0.17			
Smoking (vs. never)	1.42	0.95–2.21	0.09			
Tumor size^†^	1.60	1.44–1.78	<0.001	1.48	1.29–1.69	<0.001
LN metastasis (vs. absent)	4.35	2.87–6.58	<0.001	1.90	1.21–2.97	0.005
Pleural invasion (vs. absent)	2.03	1.35–3.04	0.001	0.91	0.59–1.42	0.68
Lymphovascular invasion (vs. absent)	4.47	2.98–6.72	<0.001	2.50	1.59–3.93	<0.001
Solid or micropapillary (vs. absent)	5.91	3.53–9.88	<0.001	3.10	1.76–5.46	<0.001
SUVmax^†^	1.07	1.04–1.09	<0.001	1.01	0.97–1.04	0.62
S/MP5 vs. S/MP−						
Age^†^	1.05	1.02–1.08	0.003	1.03	0.99–1.06	0.15
Male sex (vs. female)	0.73	0.43–1.26	0.26			
Smoking (vs. never)	1.34	0.77–2.31	0.30			
Tumor size^†^	1.64	1.42–1.90	<0.001	1.54	1.28–1.86	<0.001
LN metastasis (vs. absent)	5.86	3.28–10.46	<0.001	2.27	1.18–4.36	0.01
Pleural invasion (vs. absent)	1.87	1.08–3.25	0.03	0.75	0.41–1.38	0.35
Lymphovascular invasion (vs. absent)	4.49	2.55–7.89	<0.001	2.68	1.41–5.10	0.003
Solid or micropapillary (vs. absent)	4.98	2.81–8.82	<0.001	3.20	1.73–5.90	<0.001
SUVmax^†^	1.07	1.04–1.10	<0.001	1.02	0.98–1.06	0.40
Stage IA, S/MP5 vs. S/MP−						
Age^†^	1.04	0.99–1.10	0.14			
Male sex (vs. female)	2.79	1.01–7.67	0.05	2.18	0.66–7.19	0.20
Smoking (vs. never)	2.52	0.94–6.77	0.07			
Tumor size^†^	2.05	0.89–4.71	0.09			
Lymphovascular invasion (vs. absent)	8.47	2.12–26.39	<0.001	3.17	0.87–11.57	0.08
Solid or micropapillary (vs. absent)	4.45	1.67–11.89	0.003	3.10	1.03–9.34	0.04
SUVmax^†^	1.15	1.05–1.27	0.001	1.09	0.99–1.20	0.07
Stage IB‐IIIA, S/MP5 vs. S/MP−						
Age^†^	1.02	0.98–1.07	0.31			
Male sex (vs. female)	0.80	0.38–1.70	0.57			
Smoking (vs. never)	0.91	0.42–1.96	0.81			
Tumor size^†^	1.48	1.16–1.88	0.002	1.59	1.15–2.20	0.01
LN metastasis (vs. absent)	4.68	2.09–10.43	<0.001	3.26	1.16–9.17	0.03
Pleural invasion (vs. absent)	0.60	0.29–1.28	0.19			
Lymphovascular invasion (vs. absent)	2.73	1.25–5.98	0.01	1.48	0.54–4.01	0.45
Solid or micropapillary (vs. absent)	3.80	1.70–8.50	0.001	3.15	1.17–8.47	0.02
SUVmax^†^	1.07	1.00–1.14	0.05	1.05	0.97–1.14	0.22

CI, confidence interval; HR, hazard ratio; HRs, 95% CIs and their corresponding *P*‐values were calculated using Cox proportional hazard models; S/MP+, solid or micropapillary subtype present; S/MP−, both solid and micropapillary subtype absent; S/MP5, solid or micropapillary subtype proportion ≤ 5%; SUVmax, maximal standardized uptake value.

^†^ As continuous variable.

We further evaluated the clinicopathological features and clinical outcomes related to S/MP subtype in stage IA patients (*n* = 251). The S/MP subtype was present in 31.1% (78/251) of patients with stage IA disease, and 19.1% (48/251) had S/MP proportion of 5% or less (Table 3). For this analysis, we compared the S/MP5 and S/MP− groups to investigate whether the minimal presence of the S/MP subtype remains a critical predictor of poor surgical outcomes in stage IA. The tumor size was significantly larger in the S/MP5 group (2.0 [1.0–2.9] cm) than in the S/MP− group (1.5 [0.6–3.0] cm; *P* = 0.001). Lymphovascular invasion was significantly more frequent in the S/MP5 group (12.5% vs. 2.9%, *P* = 0.02). PET showed that the S/MP5 group had significantly higher ^18^F‐FDG uptake than the S/MP− group (SUVmax, 5.3 [1.6–22.5] vs. 2.6 [1.2–21.9], *P* = 0.001). The S/MP5 group showed significantly worse FFR than the S/MP− group in stage IA (Fig 1e, *P* = 0.001); however, the difference in DSS did not reach statistical significance (Fig SS1c, *P* = 0.10). In a multivariate analysis, the S/MP subtype (S/MP proportion of ≤5%) remained the only significant risk factor of recurrence (aHR = 3.10, 95% CI: 1.03–9.34, *P* = 0.04, Table 2). In addition, when we conducted a multivariate analysis in stages IB–IIIA, the S/MP subtype (S/MP proportion of ≤5%) was a significant independent risk factor of recurrence (aHR = 3.15, 95% CI: 1.17–8.47, *P* = 0.02), as well as tumor size and lymph node metastasis.

**Table 3:** Comparison between S/MP− and S/MP5 in stage IA

Characteristics	S/MP−*n* = 173	S/MP5*n* = 48	*P‐*value
Tumor size, cm	1.5 (0.6–3.0)	2.0 (1.0–2.9)	0.001
T stage			<0.001
T1a (≤1 cm)	35 (20.2)	1 (2.1)	
T1b (>1 cm but ≤2 cm)	94 (54.3)	26 (54.2)	
T1c (>2 cm but ≤3 cm)	44 (25.4)	21 (43.8)	
Tumor invasion			
Lymphatic	4 (2.3)	4 (8.3)	0.07
Vascular	1 (0.6)	2 (4.2)	0.12
Lymphovascular	5 (2.9)	6 (12.5)	0.02
Genetic mutation^†^			
*EGFR*	60/121 (49.6)	14/35 (40.0)	0.32
*ALK*	3/134 (2.2)	1/37 (2.7)	0.87
PD‐L1 expression^†^			
PD‐L1 ≥1%	22/29 (75.9)	8/9 (88.9)	0.65
PD‐L1 ≥50%	3/29 (10.3)	2/9 (22.2)	0.36
SUVmax^†^	2.6 (1.2–21.9)	5.3 (1.6–22.5)	0.001
Recurrence	8 (4.6)	8 (16.7)	0.009
Cancer‐related death	2 (1.2)	2 (4.2)	0.21

Data are presented as medians (range) or n (%).

*ALK*, anaplastic lymphoma kinase; *EGFR*, epidermal growth factor receptor; PD‐L1, programmed death ligand 1; S/MP−, both solid and micropapillary subtype absent; S/MP5, solid or micropapillary subtype proportion ≤ 5%; SUVmax, maximal standardized uptake value.

^†^ Values were calculated among patients for whom data were available.

**Figure 2:**
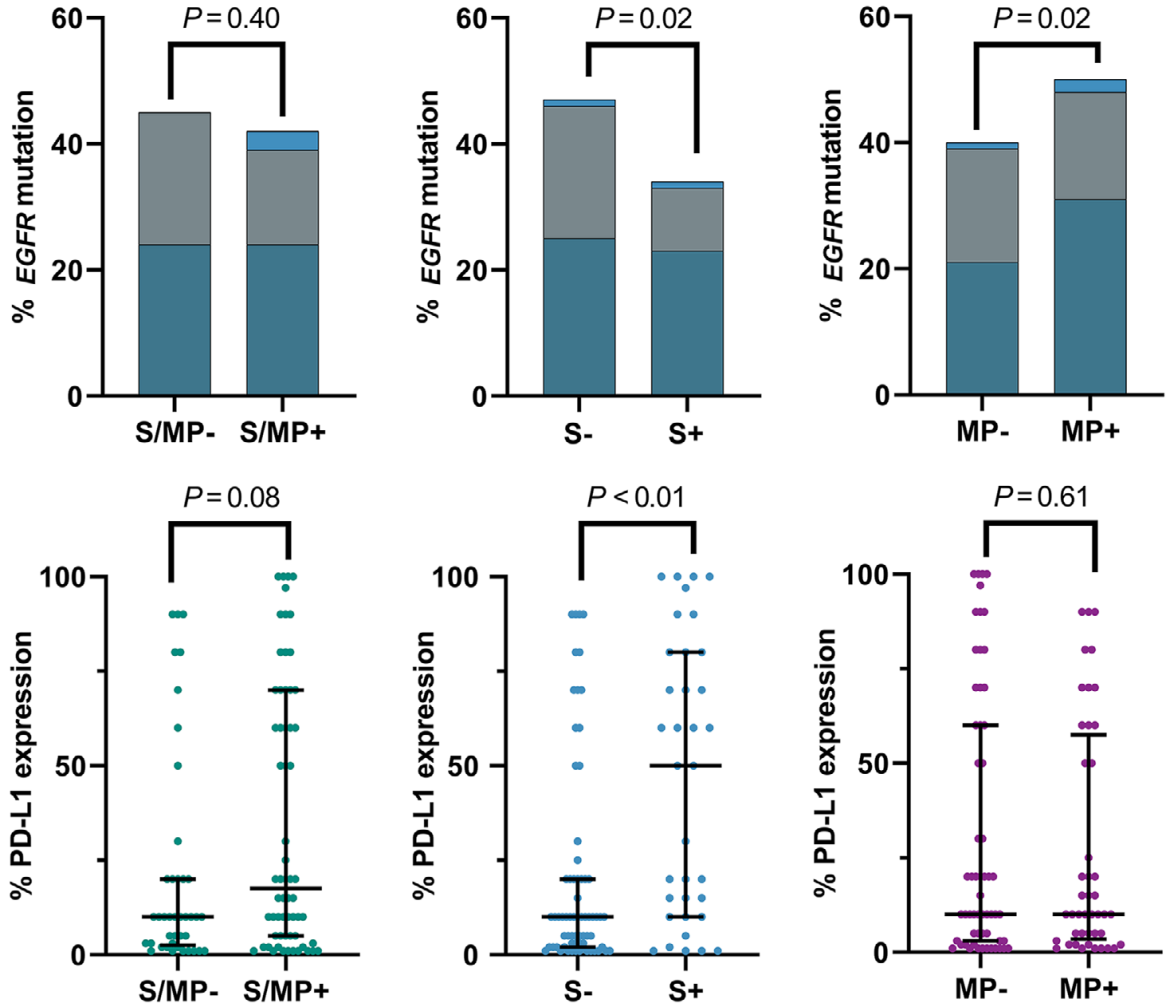
Comparison of (**a**) the frequency of *EGFR* mutation 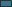, Exon 19; 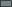, Exon 21; 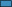, Others and (**b**) PD‐L1 expression level between solid and micropapillary subtypes. *P*‐values by χ^2^ tests (**a**), by Mann‐Whitney U test (**b**). Each dot represents one patient. Error bars represent the median ± quartile rage.

Next, we investigated whether there were different molecular features between tumors with S subtype and tumors with MP subtype (Fig 2). *EGFR* mutations were present in 51.1% of tumors in the group with the MP subtype (MP+) and in 33.9% of tumors in the group with the S subtype (S+). The MP+ group had a significantly higher frequency of *EGFR* mutation than the MP− group, whereas the S+ group had significantly lower frequency of the mutation than the S− group (*P* = 0.02 for both comparisons, Fig 2a). The frequency of *ALK* rearrangement was 6.2% in both the S+ (7/113) and MP+ groups (9/145). The PD‐L1 expression level (%) in the S/MP+ group was slightly higher than that in the S/MP− group, although the difference was not significant (*P* = 0.08, Fig 2b). Interestingly, the S+ group had a significantly higher level of PD‐L1 expression than the S− group (*P* < 0.001), whereas there was no significant difference between the MP+ and MP− groups.

## Discussion

Since the introduction of the IASLC/ATS/ERS classification of lung adenocarcinoma, the S/MP subtype has been regarded as an indicator of poor surgical outcomes. However, the clinical relevance of the minimal presence of the S/MP subtype in surgically resected specimens of lung adenocarcinoma has not as yet been clarified. In this study, we demonstrated that the minimal presence of the S/MP component was significantly associated with aggressive clinicopathological features and poor outcomes even in stage IA lung adenocarcinoma. Our results add to the evidence that the S/MP subtype is a strong risk factor of poor surgical outcome, and in particular suggests that even the minimal presence of S/MP subtype should be taken into account in the risk assessment for adjuvant chemotherapy, even in the earliest stages.

Resected pulmonary adenocarcinomas often comprise heterogeneous mixtures of multiple subtypes. Studies have reported that the prevalence among lung adenocarcinoma ranges from 16.9%–47.5% for the S subtype, 8.4%–50.1% for the MP subtype,^9^, ^11^, ^13^, ^14^, ^15^, ^16^ and 27.7%–83.0% for the combined S/MP subtype,^10^, ^12^ although the definition of the presence of a subtype in these studies varies: ≥1%,^11^, ^12^, ^13^, ^15^ ≥5%,^10^, ^14^, ^16^ or >5%,^9^ respectively. In our lung adenocarcinoma cohort, the prevalence by a ≥ 1% cutoff was 25.9% for the S subtype, 32.4% for the MP subtype, 48.8% for the S/MP subtype, and in particular, 25.5% of all patients had an S/MP proportion of ≤5%. Taken together, it is very common that the S/MP subtype is identified in a very small proportion of lung adenocarcinoma resection specimens. Therefore, it is necessary to clarify whether lung adenocarcinoma containing minimal S/MP subtype has a significantly poor prognosis. However, to our knowledge, only a few studies have evaluated the clinical outcomes of the minimal MP subtype only, and the results are limited.^13^, ^17^ In this study, S/MP+ was significantly associated with worse FFR and worse DSS in patients after complete resection than S/MP−. In every stage group, patients with S/MP+ had a significantly worse outcome, which could exclude the confounding effect of disease stage because more patients had the S/MP component at more advanced stages. Interestingly, even S/MP5 was significantly associated with more aggressive clinical features and worse prognosis in patients than S/MP−, suggesting that the presence of minimal high‐grade lung adenocarcinoma subtype components should be regarded as a potent predictor of poor prognosis.

We further analyzed 384 patients with stage I lung adenocarcinoma to evaluate whether it is reasonable to consider patients with the S/MP subtype as high‐risk subjects in potential need of adjuvant chemotherapy in this stage. Notably, the presence or absence of the S/MP subtype defined prognostic subgroups better than the stage IA/IB classification, suggesting that the presence of the S/MP subtype may be a stronger prognostic determinant than tumor size in stage I. More importantly, when we compared S/MP5 with S/MP− in stage IA patients, the minimal S/MP subtype component was the only significant independent risk factor of recurrence in a multivariate analysis. Although some studies have evaluated the clinical significance of the S or MP subtype in stage IA patients,^11^, ^14^, ^15^, ^18^ this study is the only one that has focused on the minimal presence of the S/MP subtype. Therefore, our study is the first to present an argument that the minimal presence of the S/MP subtype in stage IA lung adenocarcinoma predicts a high risk of recurrence after surgery. In this study, adjuvant chemotherapy was significantly associated with better FFR in stage II–IIIA S/MP+ patients, although the association did not reach statistical significance in stage IB S/MP+ patients (Fig SS2). The requirement for the regimen for the earliest stages may include a low toxicity profile and good patient compliance as well as efficacy. A previous phase III trial evaluated an oral agent uracil‐tegafur for stage I adenocarcinoma and showed promising results.^19^ Future clinical trials are required to validate whether the presence of S/MP subtype can justify the use of adjuvant chemotherapy in lung adenocarcinoma, especially in stage IA.

In this study, we could not explain why S/MP+ patients had a poor prognosis compared with S/MP− patients. However, this study suggested that the S and MP subtypes may have different biological backgrounds, despite a common strong correlation with aggressive phenotypes. The S and MP subtypes showed marked differences regarding *EGFR* mutation and PD‐L1 expression which was in agreement with previous studies,^20^, ^21^, ^22^, ^23^ suggesting that the two subtypes may have different mechanisms that contribute to poor prognosis. Future studies should investigate the molecular biological mechanisms underpinning the aggressive behaviors and poor outcomes of these morphologically defined adenocarcinoma subtypes to improve survival after curative resection.

Our study has some limitations. We included relatively recent patients who underwent surgery from 2014 to early 2019, therefore the duration of follow‐up may not be sufficiently long for an evaluation of survival with respect to stage IA. Additionally, the high rate of *EGFR* mutation and the use of the most recent anticancer therapeutics for lung adenocarcinoma may have considerably prolonged patient survival after recurrence. However, because most of the recurrent cases are practically incurable, the measurement of the time to recurrence may be a reasonable method to evaluate the surgical outcome in lung adenocarcinoma. Second, because this is a single‐center retrospective study, larger studies are needed to validate the clinical significance of the minimal high‐grade adenocarcinoma subtypes, especially in stage IA.

In conclusion, the minimal presence of the S/MP subtype was significantly associated with poor outcomes in surgically resected lung adenocarcinoma, even in the earliest stage. Future clinical trials are warranted to evaluate the role of adjuvant chemotherapy in these patients. In addition, further investigations on the underlying biological mechanisms of the poor prognostic effect of those subtypes are needed.

## Disclosure

The authors declare that there no conflicts of interest.

## Supporting information


**Figure S1** Kaplan‐Meier plots for disease‐specific survival according to S/MP subtype component. (**a**) S/MP+ vs. S/MP‐ (**b**) S/MP5 vs. S/MP‐ (**c**) S/MP5 vs. S/MP‐ in stage IA. S, solid; MP, micropapillary; S/MP+, solid or micropapillary subtype present; S/MP‐, both solid and micropapillary subtype absent; S/MP5, solid or micropapillary subtype proportion of 5% or less. *P*‐values by log‐rank test.

**Figure S2** Freedom from recurrence according to adjuvant chemotherapy in stage IB–IIIA S/MP+ (**a**), stage IB S/MP+ (**b**) and stage II–IIIA S/MP+ (**c**). Adj CTx, adjuvant chemotherapy. *P*‐values by log‐rank test.
