# Supplementary figures and images for: Clinical implication of minimal presence of solid or micropapillary subtype in early‐stage lung adenocarcinoma
Source: Thorac Cancer. 2020 Nov 24;12(2):235–44. doi: 10.1111/1759-7714.13754 (PMC7812076; doi:10.1111/1759-7714.13754)

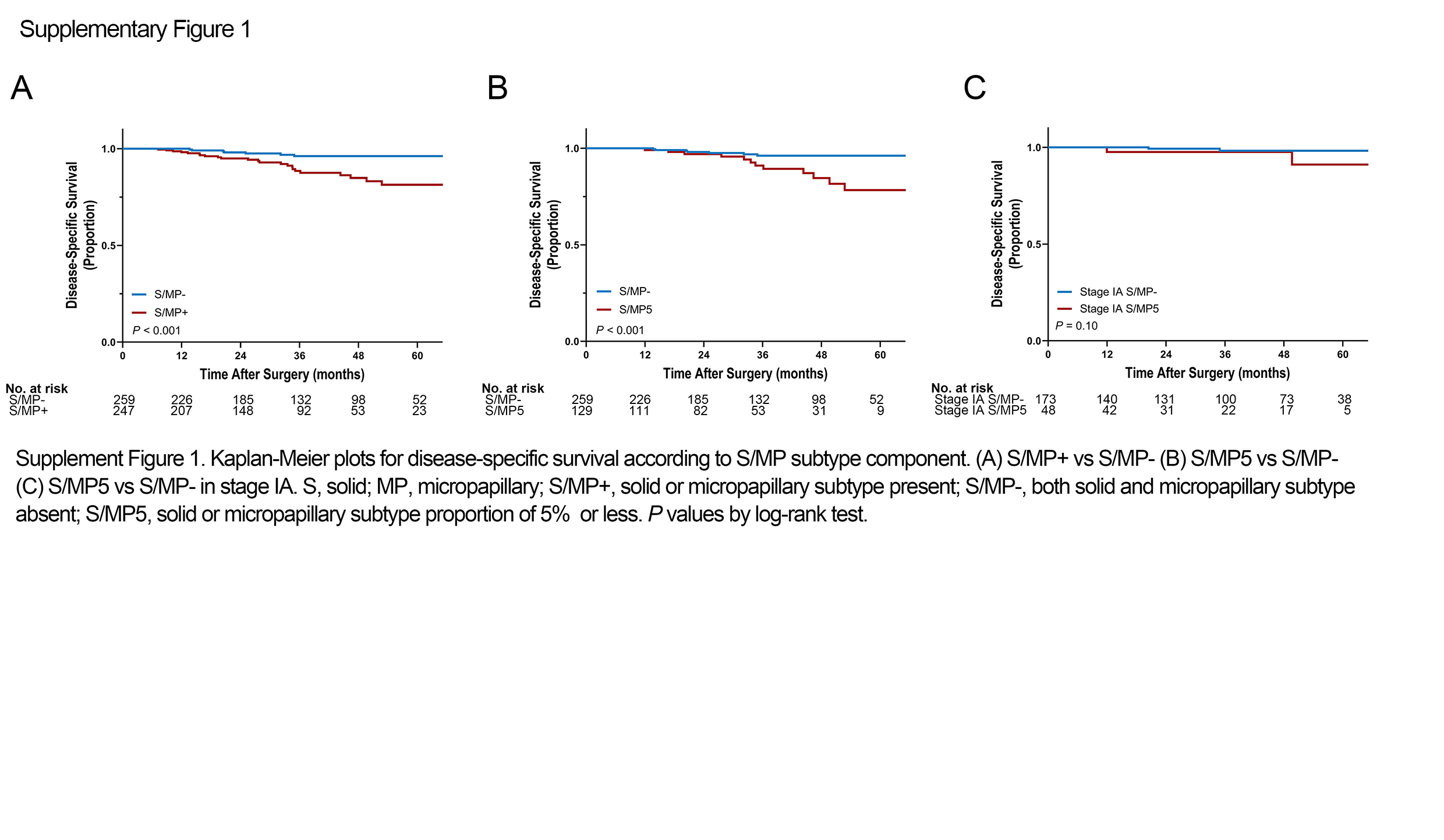

Supplement: Supplementary file 1 — Figure S1 Kaplan‐Meier plots for disease‐specific survival according to S/MP subtype component. (a) S/MP+ vs. S/MP‐ (b) S/MP5 vs. S/MP‐ (c) S/MP5 vs. S/MP‐ in stage IA. S, solid; MP, micropapillary; S/MP+, solid or micropapillary subtype present; S/MP‐, both solid and micropapillary subtype absent; S/MP5, solid or micropapillary subtype proportion of 5% or less. P‐values by log‐rank test. [file TCA-12-235-s001.png]

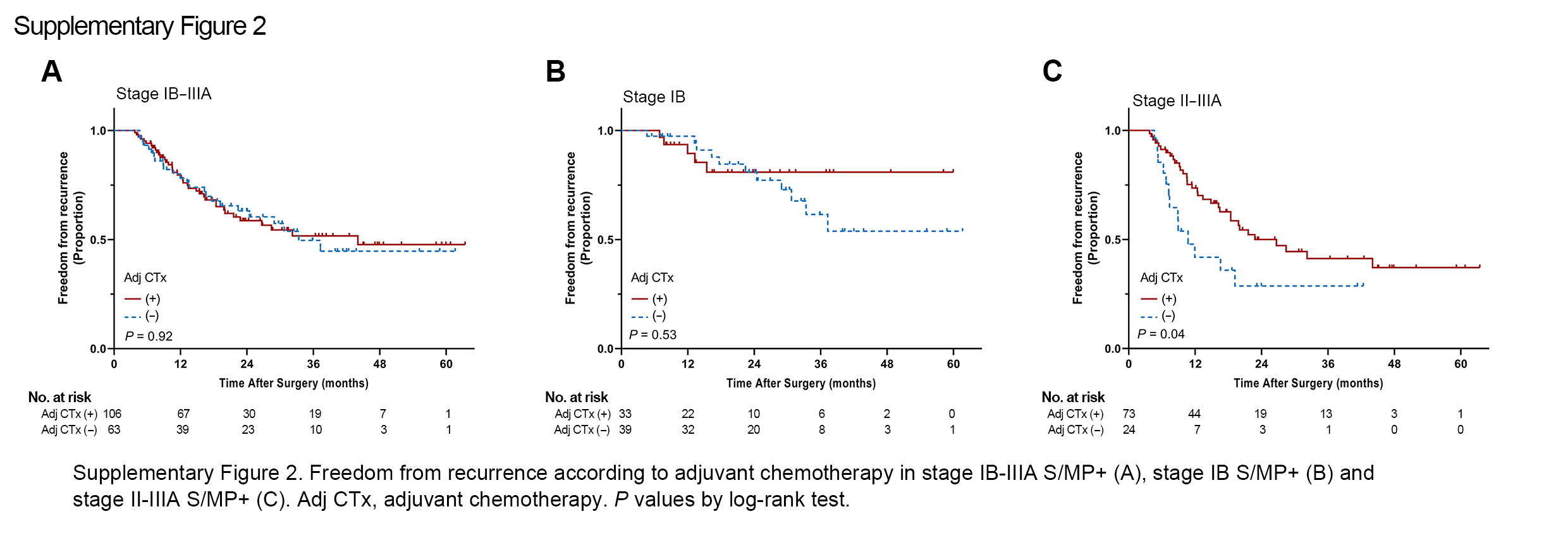

Supplement: Supplementary file 2 — Figure S2 Freedom from recurrence according to adjuvant chemotherapy in stage IB–IIIA S/MP+ (a), stage IB S/MP+ (b) and stage II–IIIA S/MP+ (c). Adj CTx, adjuvant chemotherapy. P‐values by log‐rank test. [file TCA-12-235-s002.png]
